# M-OTDR sensing system based on 3D encoded microstructures

**DOI:** 10.1038/srep41137

**Published:** 2017-01-20

**Authors:** Qizhen Sun, Fan Ai, Deming Liu, Jianwei Cheng, Hongbo Luo, Kuan Peng, Yiyang Luo, Zhijun Yan, Perry Ping Shum

**Affiliations:** 1 School of Optical and Electronic Information, Huazhong University of Science and Technology, Wuhan 430074, Hubei, P. R. China; 2 National Engineering Laboratory for Next Generation Internet Access System, Wuhan 430074, Hubei, P. R. China; 3 School of Electrical and Electronic Engineering, Nanyang Technological University, Singapore

## Abstract

In this work, a quasi-distributed sensing scheme named as microstructured OTDR (M-OTDR) by introducing ultra-weak microstructures along the fiber is proposed. Owing to its relative higher reflectivity compared with the backscattered coefficient in fiber and three dimensional (3D) i.e. wavelength/frequency/time encoded property, the M-OTDR system exhibits the superiorities of high signal to noise ratio (SNR), high spatial resolution of millimeter level and high multiplexing capacity up to several ten thousands theoretically. A proof-of-concept system consisting of 64 sensing units is constructed to demonstrate the feasibility and sensing performance. With the help of the demodulation method based on 3D analysis and spectrum reconstruction of the signal light, quasi-distributed temperature sensing with a spatial resolution of 20 cm as well as a measurement resolution of 0.1 °C is realized.

Optical time domain reflectometry (OTDR) technology is able to monitor vibration, temperature, strain and polarization mode dispersion, etc. and provides distributed fiber sensing ability which is considered as the greatest advantage of fiber sensing technology. Different sensing measurands will introduce changes in different properties of the backscattered light like polarization and phase change of Rayleigh backscattered light, intensity variation of Raman backscattered light or frequency shift of the Brillouin backscattered light. Based on different sensing mechanisms, OTDRs can be divided into polarization OTDR (P-OTDR), phase OTDR (Φ-OTDR), Raman OTDR (R-OTDR) and Brillouin OTDR (B-OTDR) etc^1^,^2^,^3^,^4^,^5^,^6^. However, the intensity ratio of the backscattered light to the probe pulse in single mode fiber for the Rayleigh backscattered light is only about −55 dB^7^ while for Brillouin backscattered and Raman light are −67.5 dB^8^ and −70 dB^9^, respectively. Hence, the signal to noise ratio (SNR) of the sensing light is too low to maintain high precision of detection and dynamic sensing due to the averaging process. In order to balance the SNR and pulse width, the spatial resolution of traditional OTDRs is only meter-level^1^,^2^,^3^,^4^,^5^,^6^. Recently, Brillouin optical time domain analysis (BOTDA) technique, adopting the pump light to enhance the signal light intensity in the sensing fiber^4^, is developed. With a novel pulse encoding scheme^10^, spatial resolution up to 2 cm can be realized along the 2 km long fiber. However, BOTDA system always employs loop structure which is not convenient for practical applications. The optical frequency domain reflectometry (OFDR) technology has also been utilized to interrogate the sensing information in frequency domain^11^,^12^. Spatial resolution of 0.3 mm has been reported^13^, which is higher than other schemes, but the sensing length is limited by the coherence length of the laser source and the sensing performance is deteriorated by the accumulated phase noise.

Fiber Bragg gratings (FBG) based quasi-distributed sensing network is another popular type of distributed sensor. Assisted with wavelength division multiplexing (WDM)^14^,^15^, resolution of sub centimeter can be achieved, but the number of sensing units as well as the sensing distance is very limited due to the small number of wavelength channels. Using the identical ultra-weak FBG as the sensing unit is referred as λ-OTDR^16^. Single detection can obtain the measurands along several kilometers long fiber^17^, and thousands of sensing points can be multiplexed^18^,^19^. However, the realized spatial resolution is limited to only 1 m^17^,^20^,^21^, owing to the single location mechanism determined by the delay time between the back reflected light pulses and the input pulse.

Fabry-perot interferometric (FPI) structure consisting of two identical ultra-weak FBGs has been reported to be frequency multiplexed^22^,^23^ and recently proved to have simultaneously wavelength and frequency encoded nature in our previous work^24^, and consequently achieve high multiplexing capacity up to 1000 along single fiber by adopting hybrid WDM and FDM techniques. Similar to the FBG, the FPI microstructure is also easy to be inscribed on the fiber through the UV exposure^24^.

In this paper, we further propose a new concept of microstructured OTDR (M-OTDR) by introducing the ultra-weak FPI microstructures with three dimensional (3D) encoded property i.e. wavelength, frequency and time, as well as 3D demodulation scheme based on the back reflected light pulses. Through addressing every sensing point in time, wavelength and frequency domains, higher spatial resolution, SNR and multiplexing capacity are expected. A proof-of-concept M-OTDR system is built to experimentally demonstrate the feasibility and sensing performance. At last, the theoretical multiplexing capacity and spatial resolution are systematically discussed.

## Results

### Sensing Principle

**Figure 1:**
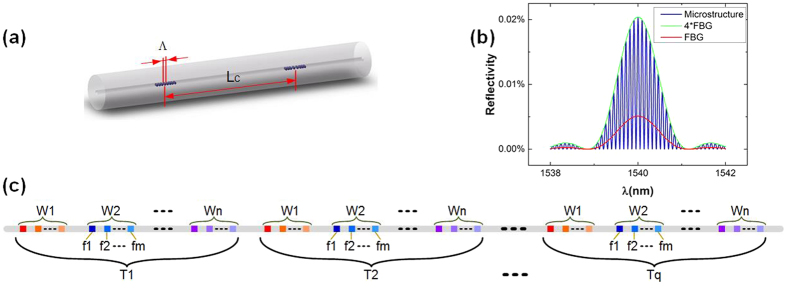
Sensing and encoding principle of M-OTDR. (**a**) An example of one microstructure; (**b**) Simulation spectra of one microstructure and one single FBG in the microstructure; (**c**) Distribution of microstructures along the sensing fiber. We define the time code as Tq, where q is the chronological order of back reflected light pulses. In every time group, we define the wavelength code as Wn where n is the size order of central wavelength of microstructure and frequency code as fm where m is the size order of free spectral range (FSR) of microstructure.

The sensing microstructure is composed of two identical ultra-weak uniform FBGs, as shown in Fig. 1(a). Λ is the grating period and *L*_*C*_ is the cavity length between the two gratings. Due to the low reflectivity, reflected light of one microstructure can be simplified as the two-beam interference of the two FBGs. When strain or temperature is applied to the microstructure, the optical path difference(OPD) between the two gratings will change, and spectrum will shift linearly because the phase matching condition must be satisfied at the peak position of the spectrum. Then the cavity length of the microstructure can be deduced:


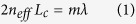


where m is a signless integral and *n*_*eff*_ is the effective index of the fiber, *λ* is the wavelength of the peaks. Tracking one peak of the spectrum can obtain the sensing information.

### Encoding Principle

During the fabrication process, two key parameters of microstructure are the Λ and the *L*_*C*_. Figure 1(b) illustrates the simulation spectra of one microstructure presented as the blue curve and one FBG presented as the red curve to investigate how the two parameters influence the microstructure. From the simulation, it can be seen that the envelope of the microstructure spectrum presented as the green curve is just four times as large as the FBG spectrum. Therefore, the central wavelength of the microstructure can be predetermined by Λ. In addition, the spectra of one microstructure will be transformed into a triangle-shaped peak after the fast Fourier transformation (FFT). The peak frequency equals to the reciprocal of the free spectral range(FSR) which is determined by the cavity length *L*_*C*_. The low reflectivity also enables large quantities of microstructures to be multiplexed in the time domain due to the low insertion loss of every sensing unit. We define the time delay time between the input pulse and reflected pulses from microstructures as *τ*. Thus, adjusting Λ, *L*_*C*_ and *τ* can encode microstructure in wavelength, frequency and time domain simultaneously. Figure 1(c) presents distribution of microstructures along the sensing fiber in M-OTDR system. Microstructures is identified through the combination of time code Tq, wavelength code Wn and frequency code fm.

When a probe light pulse is launched into the sensing fiber, microstructures with same time code are firstly located and distinguished through the time delay of received pulses roughly. Demodulation module analyzing the spectrum to obtain the wavelength and frequency code, which can be used to locate every single microstructure. In this paper, the accurate spectrum analysis is completed through the tunable FP filter demodulation scheme. After the spectrum analysis, different microstructures with different wavelength code can be distinguished. At last, we apply a filtering algorithm to the obtained spectrum at different wavelength section, spectrum of every microstructure can be reconstructed through the filtering process and calculate the spectrum shift of every microstructure to get the sensing information. Furtherly, the 3D code of every microstructure, which can be used to locate the sensing position is obtained during the demodulation process.

Owing to this 3D encoding mechanism, microstructures spaced closely to each other can be further separated through wavelength and frequency code in the time group, resulting in great improvement of the spatial resolution. And the capacity of M-OTDR will also be higher than the traditional λ-OTDR due to the hybrid multiplexing mechanism.

### Experiment setup

**Figure 2:**
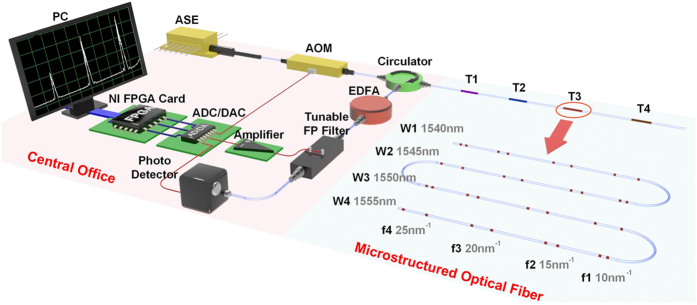
Experiment setup.

To demonstrate the feasibility and performance of the M-OTDR sensing system, a proof-of-concept system including 64 sensing microstructures along the 120 m long fiber is built. The setup of the sensing system is illustrated in Fig. 2, which consists of the central office (CO) and the microsructured optical fiber (MOF). In the CO, an amplified spontaneous emission (ASE) light source emits the broad band light ranging from 1535 nm to 1565 nm, the acoustic optical modulator (AOM) driven by NI FPGA card modulates the continuous light into pulses with width of 200 ns and then the probe light pulse is launched into MOF through a circulator. The reflected pulse train from the MOF goes through the circulator and is amplified by the erbium doped fiber amplifier (EDFA) for sensing power boost. Then, a tunable FP filter (TFF) with 16 pm bandwidth, driven by the NI FPGA card through amplifier to magnify and stabilize the voltage signal from digital to analog converter(DAC) is set to the desired wavelength to filter and suppress the unconcerned light noise induced by EDFA. The back reflected light is then received by avalanche photo detector (APD) and sampled by the analog to digital converter(ADC) which is also controlled by the NI FPGA card to ensure the synchronization. Light intensity data at central wavelength of TFF for different time groups is obtained. Repeat the above operations at different wavelength of TFF, spectrum of every time group can be reconstructed.

The MOF in experiment is composed of 64 microstructures with 4 wavelength*4 frequency*4 time codes. The four time groups are T1, T2, T3 and T4 separated by 25 m long delay fiber. In time group T3, distribution map of 16 microstructures is illustrated in the inset of Fig. 2 in which microstructures are divided into four wavelength groups centered at 1540 nm(W1), 1545 nm(W2), 1550 nm(W3) and 1555 nm(W4), respectively, and each wavelength group contains 4 microstructures with cavity length of 8 mm, 12 mm, 16 mm and 20 mm corresponding to 4 frequencies of 10 nm^−1^(f1), 15 nm^−1^(f2), 20 nm^−1^(f3) and 25 nm^−1^(f4), respectively. In each time group, microstructures are distributed at equal distance of 20 cm. Reflectivity of single FBG is 2% with 3 dB bandwidth of 2 nm.

### Time code analysis

**Figure 3:**
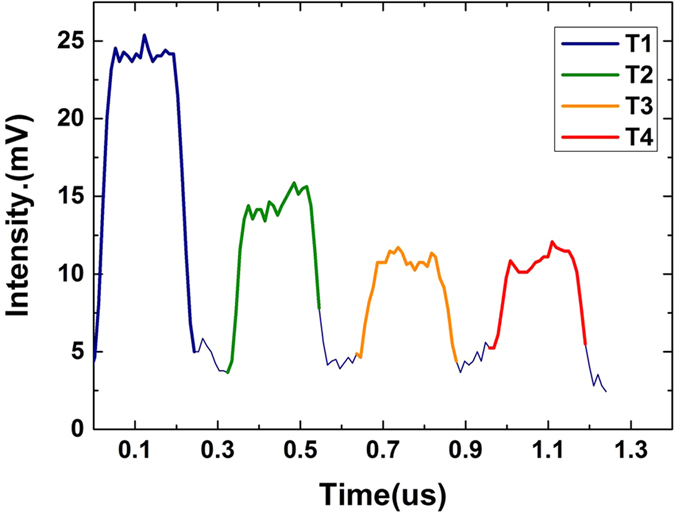
Back reflected pulse train when the central wavelength of tunable FP filter is set at 1550.2 nm.

Calculating the delay time between the back reflected pulses and the emission pulse can extract the time code at first. Back reflected light received at 1550.2 nm is illustrated in Fig. 3. It can be seen that one trace received at certain wavelength takes about 1.2 us. Intensity data of different time groups are easy to distinguish. Time delays for the four pulses indicate that the distance between the four time groups and the light source are 2 m, 37 m, 68 m, 100 m respectively. Intensity data at other wavelength points for different time groups is received as well.

### Wavelength code analysis

**Figure 4:**
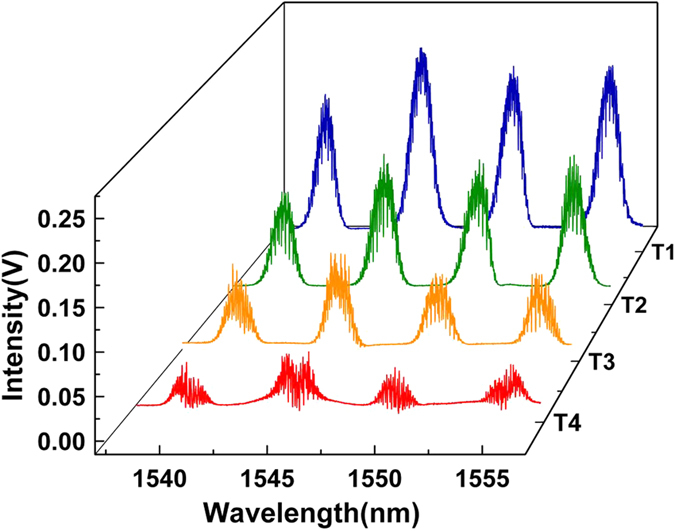
Wavelength code analysis for the four time groups with time code T1, T2, T3 and T4.

To get the spectrum of each time group, we obtain the wavelength data by inquiring driving voltage on TFF. However, the relationship between voltage of the driving PZT and operating wavelength of TFF is slightly nonlinear due to hysteresis of PZT^25^,^26^. To get high demodulation accuracy, we test the actual filtering wavelength and the applied driving voltage on the TFF through interrogating the light from a tunable laser, and then use the polynomial fitting to obtain the accurate relationship. Specifically, with a 3-order polynomial fitting as follows, we get optimized root-mean-square error (RMSE) of only 0.8%:


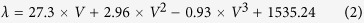


where *λ* is the wavelength, V is the driving voltage at DAC. In this way, the driving voltage is translated into wavelength accurately, and the minimum voltage change of the DAC is 3 × 10^−5^ V corresponding to minimum wavelength interval of 1 pm. Through changing the driving voltage within one period, i.e. scanning the wavelength from 1538 nm to 1558 nm, the spectrum of the microstructures in every time group can be reconstructed as displayed in Fig. 4. It is clear that there are four wavelength channels centrally locating at 1540 nm, 1545 nm, 1550 nm and 1555 nm for each time group, which are identical with the designed wavelength of the microstructures.

### Frequency code analysis

**Figure 5:**
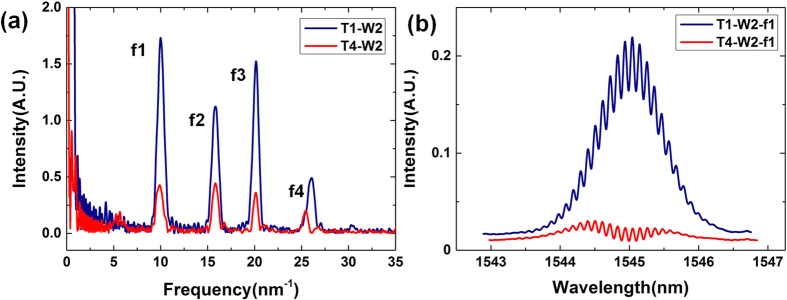
(**a**) Frequency analysis of the frequency groups with code T1-W2 and T4-W2; (**b**) Demodulated spectra of two microstructures with code T1-W2-f1 and T4-W2-f1.

To obtain the accurate position and measurand change of every sensing unit, spectra of the microstructures with the same time code and wavelength code should be further processed. Take the two frequency groups with code T1-W2 and T4-W2 for example, we apply the FFT transformation to the two peaks. The FFT frequency spectrums are illustrated in Fig. 5(a). It is obvious that there are four frequency channels in consistent with the designed parameters approximately. In addition, each frequency peak represents for one single microstructure. And the 3D code of every microstructure is obtained, which can identify the sensing unit uniquely.

### Spectrum reconstruction of every microstructure

We choose microstructure with code T1-W2-f1 and T4-W2-f1 as examples for spectrum reconstruction. After filtering process of T1-W2 and T4-W2, the reconstructed spectra are depicted in Fig. 5(b). Due to the mono frequency characteristic of the microstructure, the white noise in the original spectrum is well suppressed in the filter process and the interference fringes are clear and suitable for sensing demodulation. Although SNR of spectrum in time group T4 worsen greatly compared with T1 due to the relatively high reflectivity of single microstructure in the experiment, the interference fringe of microstructure T4-W2-f1 is still clear and suitable for later process.

We calculate the wavelength shift to obtain the sensing information. At first, we take one demodulated spectrum as the reference. Then we apply the cross correlation between the subsequent demodulated spectrum and reference spectrum to evaluate the relative wavelength shift. As a result, the quasi-distributed fiber sensing based on the MOF can be realized.

### Temperature experiment

**Figure 6:**
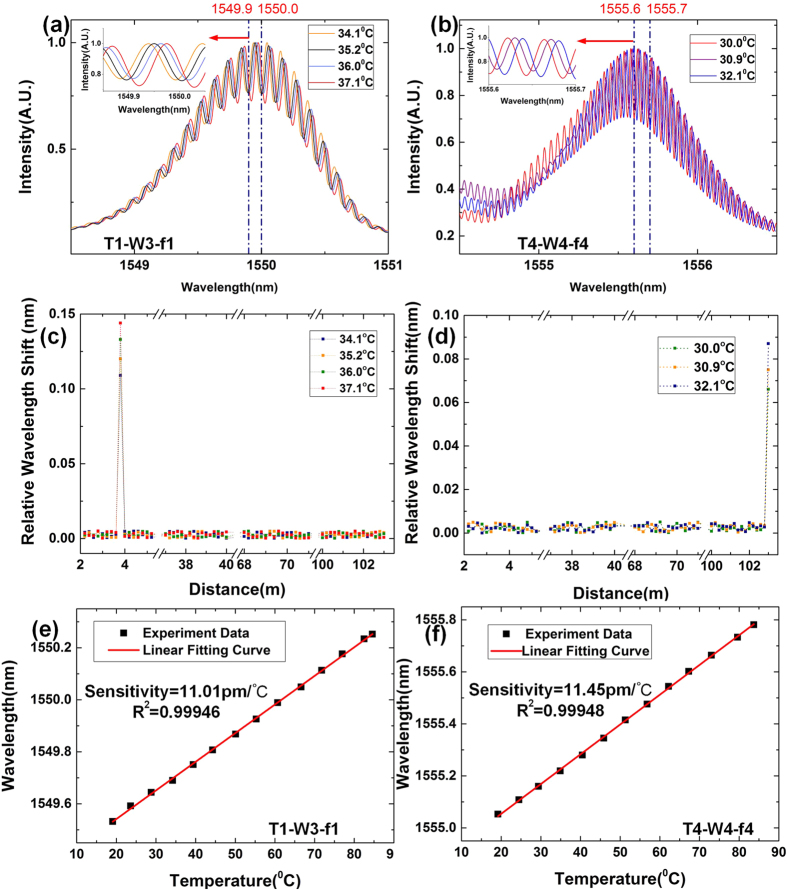
Temperature test of M-OTDR. (**a**) Spectrums of microstructure T1-W3-f1 when temperature changes at the step of 1 °C. (**b**) Spectrums of microstructure T4-W4-f4 when temperature changes at the step of 1 °C. (**c**) Relative wavelength shift of the 64 sensing points when temperature at T1-W3-f1 changes. (**d**) Relative wavelength shift of the 64 sensing points when temperature at T4-W4-f4 changes. (**e**) Tracked peak wavelength of microstructure T1-W3-f1 changes with the temperature. (**f**) Tracked peak wavelength of microstructure T4-W4-f4 changes with the temperature.

Here, we conducted a temperature experiment on the system to test the sensing performance. Two microstructures with code: T1-W3-f1 and T4-W4-f4 are chosen to put on the thermal energy converter (TEC) for accurate control of temperature change. Figure 6(a) illustrates the spectrum of microstructure T1-W3-f1 when temperature changes from 34 °C to 37 °C. As we can see that the spectrum shifts linearly with the temperature change. Spectrum of microstructure T4-W4-f4 also shifts linearly when environmental temperature changes from 30.0 °C to 32.1 °C as depicted in Fig. 6(b). Taking demodulated spectrum at room temperature of 24.2 °C as the reference, the relative wavelength shifts for all the 64 microstructures are recorded and we locate the microstructure through the 3D code. The wavelength shifts of the 64 sensing points whose positions are obtained through the 3D code are shown in Fig. 6(c) and (d). The sensing unit influenced by the TEC is easy to be distinguished while wavelength shift of other sensing points remain unchanged. Since the spatial interval between two adjacent sensing points is 0.2 m, 0.2-meter resolution is demonstrated.

At last, we change the TEC temperature from 20 °C to 85 °C with the step about 5 °C. We record the wavelength of one fringe peak in the spectrum with the temperature change. The measurement results of microstructure T1-W3-f1, T4-W4-f4 are depicted in Fig. 6(e) and (f) respectively. It is obvious that the microstructure has a good linear relationship with the temperature change. The fitting results shows that the microstructure T1-W3-f1 has sensitivity of 11.01 pm/°C and the microstructure T4-W4-f4 has sensitivity of 11.45 pm/°C. With the spectrum resolution of 1 pm, the sensing system has a temperature resolution of about 0.1 °C. Since the microstructure T4-W4-f4 locates at the far end of the sensing fiber, the uniformity of the sensing results of two microstructures demonstrates the ability to demodulate the 64 sensing points. In addition, there are 20000 wavelength samples in total when the spectrum resolution is 1 pm and the bandwidth is 20 nm. Considering the processing time for each wavelength sample is 1.2 us at least, the system demodulation time is about 24 ms.

## Discussions

The 3D encoding method enables the M-OTDR system achieve the high spatial resolution and high capacity simultaneously. To investigate the theoretical capacity of M-OTDR, we define that the number of wavelength, frequency and time channel are N, M and Q respectively. N is limited by the bandwidth of light source and dynamic range of the sensing unit. Assuming that the light source and the demodulation system have a bandwidth of 80 nm while the working bandwidth of the microstructure is less than 5 nm, N will be greater than 16.

For the frequency channel, M is decided by the maximum frequency and the minimum frequency interval. The maximum frequency is limited by the sampling interval of the analyzing equipment according to the Nyquist Sampling theory. The demodulation system in our experiment interrogates the sensing fiber with a sampling interval of 1 pm, then the maximum frequency will be 500 nm^−1^. As for the minimum frequency interval, the crosstalk between different frequency components is the one constraint factor. The FFT spectrum of microstructure is the triangle-shaped peaks^27^ and the bandwidth of one FFT frequency peak is given by equation (3).


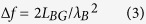


where Δ*f* is the bandwidth of the FFT frequency peak, *L*_*BG*_ is the grating length. *λ*_*B*_ is the Bragg wavelength. Now we are able to inscribe microstructure with grating length less than 1 mm on the fiber. Therefore, we can calculate that the minimum bandwidth of frequency spectrum is 1.3 nm^−1^ while the central wavelength is assumed to be 1550 nm. To avoid crosstalk, M will be 380 at maximum. Although the harmonics of different frequency channels will introduce noise to certain extent, the effect on calculating the spectrum shift of microstructure can be neglected due to two reasons. One is the high order harmonic component is very small, and the other is that there is no fixed phase relationship between different order harmonic components.

For the number of time channels, once the reflectivity of one microstructure is fixed, the greater M means the higher reflectivity of each time group which will reduce Q sharply. Therefore, there is a trade-off between M and Q. And because of the short coherent length of broad band light source, the total reflectivity of each time group at one wavelength channel will be the superposition of every single microstructure and can be expressed as:


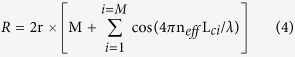


where r is the reflectivity of one single FBG, L_*ci*_ is the cavity length of the ith microstructure. We can deduce that the maximum reflectivity of one time group is:


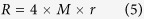


Moreover, R is also limited by the multi-path reflection between different time domains, which should satisfy the following relationship^17^


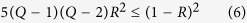


From equations (5) and (6) we can deduce that:


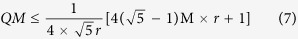


It is obvious that low reflectivity of every single microstructure is desired to achieve high capacity. With the help of the draw tower technique, the minimum peak reflectivity of every FBG could be as lower as −40 dB^28^. Then, the maximum value of Q*M will be 1140 when r = −40 dB, m = 380. Thus total capacity of M-OTDR sensing system will be above 18000. With the help of draw-tower technique^28^, it is easy to provide enough sensing points in M-OTDR sensing system for different applications.

In addition, sensing points encoded in different wavelength code or frequency code in every time group can be distinguished in spectrum. The minimum spatial resolution will be the microstructure length which can reach several millimeters.

In summary, benefitting from the high SNR, 3D encoding ability and large multiplexing capacity of the microstructure, the M-OTDR can provide the advantages of high spatial resolution, high measurement accuracy, long sensing distance and flexible system configuration for different applications.

## Conclusion

In conclusion, a novel 3D encoded microstructured OTDR sensing system for improvement in the SNR, capacity and spatial resolution is demonstrated. Microstructure consisting of two identical ultra-weak FBGs can be encoded in time, wavelength and frequency simultaneously. A demodulation platform based on TFF is constructed to evaluate the 3D code of every microstructure. A proof of concept MOF with 4 wavelength*4 frequency*4 time codes is demodulated successfully. Temperature experiment has shown that a sensitivity of about 11 pm/°C and a spatial resolution of 0.2 m is demonstrated. In addition, through the theoretical analysis, M-OTDR sensing system is proved to have the potential to achieve high capacity up to 18000 and high spatial resolution of only several millimeters. With appropriate configuration of the microstructures, M-OTDR system is flexible to adapt to different sensing areas.

## Additional Information

**How to cite this article**: Sun, Q. *et al*. M-OTDR sensing system based on 3D encoded microstructures. *Sci. Rep.* **7**, 41137; doi: 10.1038/srep41137 (2017).

**Publisher's note:** Springer Nature remains neutral with regard to jurisdictional claims in published maps and institutional affiliations.
